# CDH1 and IL1-beta expression dictates FAK and MAPKK-dependent cross-talk between cancer cells and human mesenchymal stem cells

**DOI:** 10.1186/s13287-015-0123-0

**Published:** 2015-07-24

**Authors:** Mashael Al-toub, Radhakrishnan Vishnubalaji, Rimi Hamam, Moustapha Kassem, Abdullah Aldahmash, Nehad M. Alajez

**Affiliations:** Stem Cell Unit, Department of Anatomy, College of Medicine, King Saud University, Riyadh, 11461 Kingdom of Saudi Arabia; KMEB, Department of Endocrinology, University Hospital of Odense and University of Southern Denmark, 5000 Odense C, Denmark; DanStem, Danish Stem Cell Center, Panum Institute, University of Copenhagen, 2200 Copenhagen N, Denmark; Prince Naif Health Research Center, King Saud University, Riyadh, 11461 Kingdom of Saudi Arabia

## Abstract

**Introduction:**

Tumor microenvironment conferred by stromal (mesenchymal) stem cells (MSCs) plays a key role in tumor development, progression, and response to therapy. Defining the role of MSCs in tumorigenesis is crucial for their safe utilization in regenerative medicine. Herein, we conducted comprehensive investigation of the cross-talk between human MSCs (hMSCs) and 12 cancer cell lines derived from breast, prostate, colon, head/neck and skin.

**Methods:**

Human bone marrow-derived MSC line expressing green fluorescence protein (GFP) (hMSC-GFP) were co-cultured with the following cancer cell lines: (MCF7, BT-20, BT-474, MDA-MB-468, T-47D, SK-BR-3, MDA-MB-231, PC-3, HT-29, MDA-MB-435s, and FaDu) and changes in their morphology were assessed using fluorescent microscopy. For cellular tracking, cells were labeled with Vybrant DiO, DiL, and DiD lipophilic dyes. Time-lapse microscopy was conducted using Nikon BioStation IM-Q. Stable expression of mCherry, and luciferase genes was achieved using lentiviral technology. IL1-Beta neutralizing experiments were conducted using soluble recombinant IL-1R (srIL-1R). Changes in gene expression in sorted hMSCs were assessed using Agilent microarray platform while data normalization and bioinformatics were conducted using GeneSpring software.

**Results:**

We observed a dynamic interaction between cancer cells and hMSCs. High CDH1 (E-cadherin) and low IL1-Beta expression by cancer cells promoted reorganization of hMSCs into a niche-like formation, which was dependent on direct cell-cell contact. Our data also revealed transfer of cellular components between cancer cells and hMSCs as one possible mechanism for intercellular communication. Global gene expression analysis of sorted hMSCs following co-culturing with MCF7 and BT-20 cells revealed enrichment in signaling pathways related to bone formation, FAK and MAPKK signaling. Co-culturing hMSCs with MCF7 cells increased their growth evidenced by increase in Ki67 and PCNA staining in tumor cells in direct contact with hMSCs niche. On the other hand, co-culturing hMSCs with FaDu, HT-29 or MDA-MB-231 cells led remarkable decline in their cell growth.

**Conclusions:**

Dynamic interaction exists between hMSCs and cancer cells. CDH1 and IL1-Beta expression by cancer cells mediates the crosstalk between hMSCs and cancer cells. We propose a model where hMSCs act as the first line of defense against cancer cell growth and spread.

**Electronic supplementary material:**

The online version of this article (doi:10.1186/s13287-015-0123-0) contains supplementary material, which is available to authorized users.

## Introduction

Carcinogenesis is a complex process that involves transformed cells interacting with the microenvironment containing extracellular matrix, carcinoma-associated fibroblasts (CAFs), pericytes, endothelial cells, and immune cells [1]. Cross-talk between transformed cells and the microenvironment contributes to tumor growth, invasion, and metastasis. Among tumor microenvironment components, growing evidence suggests that CAFs are derived from mesenchymal (stromal) stem cells (MSCs), which are multipotent stem cells present within the stroma of bone marrow and probably other organs [2]. The precise role of CAFs or MSCs in cancer development and progression is an area of intensive investigation and remains controversial (for a review see [3]). For instance, Karnoub et al. [4] reported that MSCs in a breast cancer xenograft model promoted breast cancer invasion and metastasis via the chemokine (C–C motif) ligand/C–C chemokine receptor CCL5/CCR5 cytokine network. Similarly, Liu et al. [5] reported that MSCs promoted breast cancer stem cell expansion via interleukin (IL)-6 and chemokine (C–X–C motif) ligand 7 signaling. In another study, Huang et al. [6] demonstrated that activation of caspase 3 by tumor or stroma cells triggers tumor repopulation during radiation therapy. While these reports suggest a pro-tumorigenic role for MSCs, a number of other studies revealed an anti-tumor effect of MSCs. For example, Cooke et al. [7] have shown that targeted depletion of pericytes (which are part of the MSC lineage) in vivo promoted tumor metastasis, which was mediated via hypoxia-induced epithelial to mesenchymal transition. Also, Khakoo et al. [8] have reported a strong inhibitory effect of human bone marrow-derived MSCs (hMSCs) against Kaposi sarcoma in vitro and in vivo through inhibition of AKT signaling in tumor cells. The precise role of MSCs in tumorigenicity and the conditions under which MSCs exert pro-tumor or anti-tumor effects therefore need to be determined.

In the majority of previous studies, a single or a few tumor models were studied, which limits the generalizability of their findings to other tumor models. In the present study, we conducted a comprehensive investigation to characterize the cellular and molecular phenotype of hMSCs co-cultured with 12 cancer cell lines derived from the breast, colon, prostate, head and neck, and melanoma. Our data revealed that the outcome of MSC–tumor interaction is dependent on the nature rather than the type of tumor cells and that epithelial cadherin type 1 (CDH1) and IL-1β expression by tumor cells are key factors in determining the outcome of hMSC–tumor cross-talk.

## Methods

### Cell lines and culture

Tumor cell lines used in this study (breast: MCF7, MDA-MB-231, BT-20, BT-474, MDA-MB-468, T-47D, and SKB-R3; melanoma: MDA-MB-435S; prostate: PC-3; head and neck: FaDu; and colon: HT-29 and COLO-320) were purchased from Cell Lines Service GmbH Eppelheim, (Germany) or were obtained from other sources and subsequently authenticated by Genetica DNA Laboratories, Inc. Burlington, (NC, USA). As a model for primary hMSCs, we employed a well-characterized telomerized hMSC line (hMSC-TERT) that has been created through overexpression of the human telomerase reverse transcriptase gene (TERT) [9]. The hMSC-TERT cell line expresses all known markers of primary hMSCs [10, 11] and exhibits hMSC “stemmness”, evidenced by being able to form bone and the bone marrow microenvironment following in-vivo implantation [12]. hMSC-TERT cells were also engineered to express enhanced green fluorescent protein (GFP) gene [13]. All cell lines were cultured in Dulbecco’s modified Eagle’s medium (DMEM) supplemented with 4500 mg/l d-glucose, 4 mM l-glutamine, and 110 mg/l sodium pyruvate, 10 % fetal bovine serum (FBS), 1 % penicillin–streptomycin and nonessential amino acids. The normal, nontransformed human mammary epithelial cell line (MCF10A) was maintained in universal medium (DMEM-F12 + 20 ng/ml human epidermal growth factor (EGF), 100 ng/ml cholera toxin, 0.01 mg/ml insulin, 500 ng/ml hydrocortisone, and 5 % FBS). Primary normal adipose tissue-derived MSCs (AT-MSCs) were obtained and cultured as described previously [14].

### Co-culture experiments

For co-culture experiments, hMSCs were trypsinized, counted, and seeded at 0.5 × 10^5^/well, and 1 × x10^5^ tumor cells were added to the same well in 24-well culture plates (Falcon, Franklin Lake, NJ, USA). Co-cultures were subsequently monitored and images were taken at the indicated time points using a Nikon® ECLIPSE Ti-U inverted fluorescence microscope, (Nikon, Tokyo, Japan). Cells were either imaged directly or were washed with 1× phosphate-buffered saline (PBS), followed by staining with Hoechst 33342 (10 μg/ml) in PBS for 10 minutes at 37 °C. Recombinant human IL-1β was purchased from (Invitrogen, Carlsbad, CA, USA). Focal adhesion kinase (FAK) inhibitor (PF-573228), mitogen-activated protein kinase kinase (MAPKK) inhibitor (PD98059), and cytochalasin D were purchased from Sigma (St. Louis, MO, USA) and were reconstituted in dimethyl sulfoxide (DMSO). For experiments involving IL-1β or the abovementioned small molecule inhibitors, the agents were added from day 0 of the experiment as indicated in each figure.

### Vybrant® multicolor cell labeling

Cells were harvested and suspended at a density of 1 × 10^6^/ml in serum-free culture DMEM followed by fluorescence-labeling with the relevant Vybrant® Cell-Labeling Solution (Invitrogen, Carlsbad, CA, USA) as per the manufacturer’s protocol. AT-MSCs were labeled with green fluorescent probe (DiO), while the MCF7 cell line was labeled with DiL or DiD probes. Subsequently, direct co-culturing was performed by seeding the labeled primary hMSCs and the labeled MCF7 cells into 24-well culture plates at density of 1 × 10^5^/ml/well for each cell type. Co-cultures were visualized and images were taken on the indicated days.

### Lentiviral transduction and time-lapse microscopy

Lentiviral particles encoding for mcherry or firefly luciferase were purchased from Genecopoeia Inc. (Rockville, MD, USA). Then 100,000 tumor cells were seeded in complete DMEM in a 24-well plate. Forty-eight hours later (~80 % confluency), the medium was removed and then 20 μl crude lentiviral particles in 500 μl DMEM + 5 % heat-inactivated serum (Invitrogen), 1 % penicillin–streptomycin supplemented with polybrene (8 μg/ml; Sigma) were added to the cells. Seventy-two hours later, the medium was removed and transduced cells were selected with puromycin (1 μg/ml; Sigma) for 1 week until stably-transduced cells were generated. Time-lapse microscopy was conducted using Nikon® BioStation IM-Q.

### Magnetic activated cell sorting

Direct co-culture of MCF7 (seeding number = 1.0 × 10^5^/well) or BT-20 (seeding number = 1.0 × 10^5^/well) cells and hMSCs (seeding number = 0.5 × 10^5^/well) was conducted using BD Falcon 24-well culture plates. On day 7 when the niche-like structure was visible, co-cultured cells were trypsinized from three replicas, and washed once with 1× PBS. Cells were subsequently resuspended in sorting buffer (500 ml Ca^2+^/Mg^2+^ free PBS supplemented with 2 mM ethylenediamine tetraacetic acid (EDTA) and 0.5 % bovine serum albumin (BSA)), which was subsequently filtered using a 0.22 μM filter. Sorting of hMSCs from MCF7 or BT-20 co-cultures was performed using the CD326 (EpCAM) MicroBeads kit (Miltenyi Biotec GmbH, Bergisch Gladbach, Germany) as per the manufacturer’s recommendations. Purity of sorted cells was confirmed using the BD FACSCalibur flow cytometer (BD Biosciences, San Jose, CA, USA). Sorted cells were then washed with 1× PBS and were kept at −80 °C. Cells from the same batch and passage number of hMSCs were used as control.

### Gene expression microarray

Total RNA was isolated using total RNA Purification Kit (Norgen-Biotek Corp., Thorold, ON, Canada) according to the manufacturer’s instructions. The concentrations and purity of total RNA were measured using NanoDrop 2000 (Thermo-Scientific, Wilmington, DE, USA). Extracted RNA was labeled and then hybridized to the Agilent Human SurePrint G3 Human GE 8 × 60 k microarray chip (Agilent Technologies, Santa Clara, CA, USA). All microarray experiments were conducted at the Microarray Core Facility (Stem Cell Unit, King Saud University College of Medicine, Riyadh, Saudi Arabia) as described previously [15]. Data analyses were conducted using GeneSpring GX software (Agilent Technologies) as described [16, 17]. Microarray data were deposited in the Gene Expression Omnibus [GEO:GSE70103].

### Quantification of gene expression using qRT-PCR

Expression levels of selected genes were assessed using qRT-PCR. Reverse transcription was performed on 500 ng total RNA using High Capacity Reverse Transcriptase Kit (Applied Biosystems, Foster City, CA, USA) according to the manufacturer’s specifications. The qRT-PCR was carried out using FAST-SYBR Green Master Mix (Applied Biosystems) and the ViiA™ 7 Real-Time PCR Detection System (Applied Biosystems). Primers used for gene expression analysis are presented in Additional file 1 and were either published previously or were designed using NCBI Primer-BLAST [18]. The 2∆CT value method was used to calculate relative expression of miRNAs and mRNAs [19].

### Alkaline phosphatase staining

hMSCs were co-cultured with MCF7 or HT-29. On day 7, the cells were washed in PBS, fixed in 10 mM acetone/citrate buffer (1.5:1) at pH 4.2 for 5 minutes at room temperature, and incubated with alkaline phosphatase (ALP) substrate solution (naphthol AS-TR phosphate (Sigma) prepared 1:5 in water plus 10 mg Fast red TR (Sigma), in 24 ml of 0.1 M Tris buffer, pH 9.0) for 1 hour at room temperature. Cells were rinsed with water, stored in PBS, and photographed using a Nikon® ECLIPSE Ti-U inverted fluorescence microscope.

### Immunofluorescence staining

Day 7 MCF7–MSC co-cultures were fixed with methanol/acetone 1:1 (vol/vol) for 30 minutes at −20 °C. After fixation, cells were dried for 15 minutes and rehydrated with PBS for 15 minutes. Cells were blocked with 2 % BSA (Sigma) for 1 hour, followed by incubating with rabbit primary antibodies against Ki67 (1:200, ab15580; Abcam, Cambridge, MA, USA) or Proliferating cell nuclear antigen (PCNA) (1:100, ab2426; Abcam) in blocking solution at 4 °C overnight. After removal of primary antibodies, cells were washed three times with PBS, and then Alexa Fluor® 488 conjugated goat anti-rabbit IgG (H + L) secondary antibody (1/1000, A11008; Lifetechnologies, Carlsbad, CA, USA) was added and incubated for 2 hours at room temperature. Cells were washed three times with PBS and counterstained with 4′,6-diamidino-2-phenylindole (DAPI) nuclear dye, mounted on slides in CC/Mount (C9368; Sigma), and were observed under a Nikon®ECLIPSE Ti fluorescence microscope.

### Flow cytometry

All flow cytometry experiments were conducted using the BD FACSCalibur flow cytometer (BD Biosciences).

### Cell counting

For counting tumor and hMSC cells from co-culture experiments, we employed the following strategy. Co-cultured cells were trypsinized and then the percentage of hMSC cells (GFP^+^) and tumor (GFP^–^) was determined using fluorescence-activated cell sorting (FACS). The total number of cells in the co-culture per sample was determined using an automated cell counter (Vi-Cell XR cell viability analyzer; Beckman Coulter Inc, Fullerton, CA, USA). The number of hMSCs was calculated using the following equation:

$$ \mathrm{hMSCs} = \%\ {\mathrm{GFP}}^{+} \times \mathrm{total}\ \mathrm{number}\ \mathrm{of}\ \mathrm{cells}\ \mathrm{and}\ \mathrm{the}\ \mathrm{number}\ \mathrm{of}\ \mathrm{tumor}\ \mathrm{cells} = \%\ {\mathrm{GFP}}^{\hbox{-}} \times \mathrm{total}\ \mathrm{number}\ \mathrm{of}\ \mathrm{cells}. $$

For selected experiments, the relative number of hMSCs was determined as follows. hMSCs 0.5 × 10^4^ were cultured alone or with MCF7 (0.5 × 10^3^) or HT-29 (0.5 × 10^3^) cells in Corning® polystyrene flat-bottomed 96-well TC-treated black microplates Cambridge, MA, USA. The fluorescence signal was measured using a SpectraMax/M5 fluorescence spectrophotometer plate reader (Molecular Devices Co., Sunnyvale, CA, USA USA) using the bottom well-scan mode where nine readings per well were obtained using Ex (488 nm) and Em (509 nm) spectra. For luminescence measurements, hMSCs and tumor cells were cultured as above in Nunc™ F96 MicroWell™ white plates (Thermo-Scientific, Wilmington, DE, USA), and luminescence was measured using a BioTek Synergy II microplate reader (BioTek Inc., Winooski, VT, USA) with the ONE-Glo™ Luciferase Assay System (Promega, Madison, WI, USA).

### Scratch assay

HT-29 and MCF7 cells were cultured alone or were co-cultured with hMSCs, and on day 7 the scratch assay was performed using a p200 pipette tip. The scratch area was imaged on day 0 and day 2 using 4× magnification with the Nikon® ECLIPSE Ti-U inverted fluorescence microscope. Data are representative of at least three replicates.

### Statistical analysis

Statistical analyses and graphing were performed using Microsoft Excel 2010 (Microsoft, Mountain View, CA) and Graphpad Prism 6.0 software (Graphpad Software, San Diego, CA, USA). *P* values were calculated using the two-tailed *t* test and *P* <0.05 was considered statistically significant.

## Results

### Changes in hMSC morphology when co-cultured with cancer cell lines

**Fig. 1:**
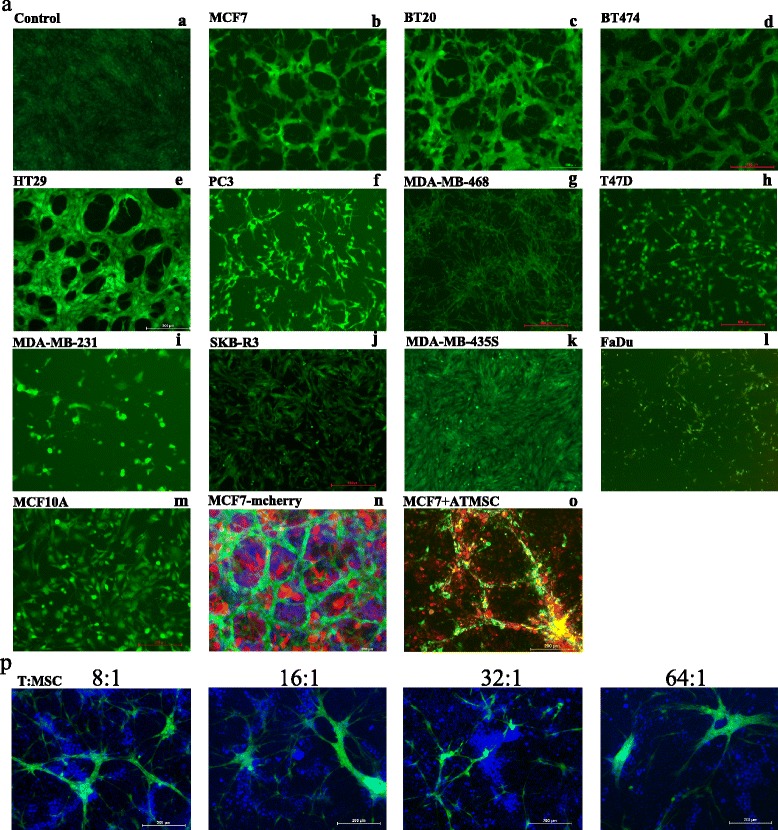
Distinct morphological changes in hMSCs co-cultured with cancer cell lines. **a**–**l** GFP-labeled hMSCs were co-cultured with the indicated tumor cell lines at a 1:2 ratio, and on day 7 of the co-culture images were taken using a Nikon® ECLIPSE Ti-U inverted fluorescence microscope (4×). Data are representative of at least three independent experiments. **m** hMSCs co-cultured with normal breast epithelial cell line (MCF10A). **n** hMSCs (GFP-labeled) co-cultured with MCF7 (mcherry-labeled) as above. Cells were stained with nuclear staining DAPI (*blue*). **o** AT-MSCs labeled with DiO (*green*) and co-cultured with MCF7 cells labeled with DiD (*red*). At day 7, photomicrographs were taken using a Nikon® ECLIPSE Ti-U inverted fluorescence microscope (magnification 4×). **p** MCF7 cells were co-cultured with hMSCs at the indicated ratios (tumor:MSCs), and on day 7 images were captured in the green channel (MSCs) using 4× magnification. DAPI was used to stain nuclei

We have previously reported significant changes in hMSC morphology when exposed to tumor-derived conditioned media, which was dependent on the nature of tumor cells [20]. However, the behavior of hMSCs cultured in direct contact with tumor cells has not been studied. We established a co-culture system consisting of GFP-labeled bone marrow-derived hMSCs in direct contact with 12 cancer cell lines representing breast (MCF7, BT-20, MDA-MB-231, MDA-MB-468, BT-474, T-47D, and SK-BR-3), colon (HT-29), prostate (PC-3), head/neck (FaDu), and skin melanoma (MDA-MB-435s). Figure 1a–o demonstrates changes in hMSC morphology in response to direct contact with cancer cell lines. One remarkable change was the cellular reorganization of hMSCs leading to the formation of a honeycomb niche-like structure when co-cultured with MCF7, BT-20, HT-29, and BT-474 (Fig. 1b, c, d, e). In contrast, hMSCs co-cultured with FaDu, T47D, and PC-3 exhibited fibroblast-like morphology and inhibition of cell growth (Fig. 1f, h, i, l). The MDA-MB-435s cell line did not affect the growth pattern of hMSCs (Fig. 1k). On the other hand, co-culturing hMSCs with nontumorigenic breast cell line MCF10A did not induce significant changes in the morphology or growth (Fig. 1m). Co-culture of GFP-labeled hMSCs and mcherry-labeled MCF7 is shown in Fig. 1n, which shows the formation of the niche-like structure by hMSCs (green), whereas the MCF7 tumor cells (red) grew in clusters surrounded by hMSCs. Similar morphological changes were observed when primary adipose-derived human stromal stem cells (AT-hMSCs) were co-cultured with MCF7 (Fig. 1o), suggesting that this observed response is conserved in hMSCs derived from different tissue compartments. The honeycomb structure of MCF7–hMSC co-cultures was also observed when hMSCs were co-cultured with MCF7 at a decreasing ratio as low as 1:64 (Fig. 1p).

### The change in hMSC morphology requires direct contact with tumor cells

**Fig. 2:**
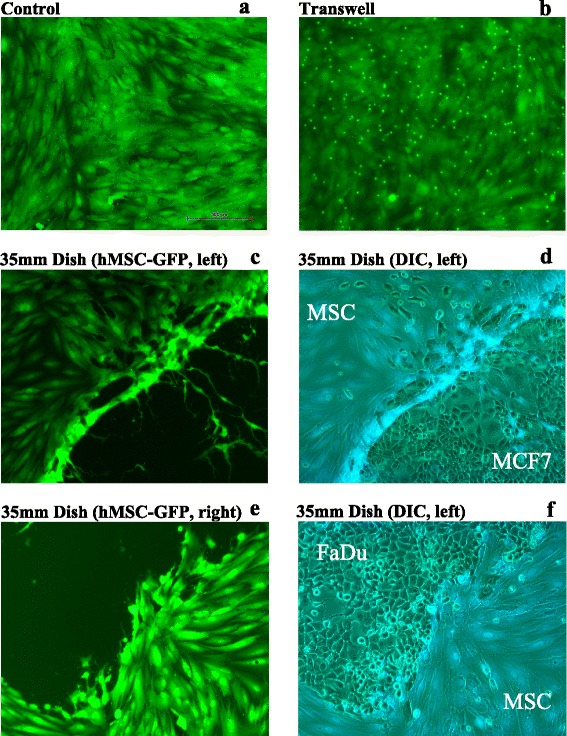
Morphological changes in hMSCs require direct contact with cancer cells. **a** control hMSCs. **b** hMSCs cultured in the upper chamber of a transwell culture system in which MCF7 cells were cultured in the lower chamber. **c**, **d** MCF7 cells were seeded on one side of 5 mm dish while FaDu cells were seeded on the opposing side of the same dish, and when cells were attached hMSCs were added to the culture: hMSCs in contact with **c**, **d** MCF7 cells or **e**, **f** FaDu cells. On day 7, photomicrographs were taken using a Nikon^®^ ECLIPSE Ti-U inverted fluorescence microscope (magnification 4×). *GFP* green fluorescent protein, *hMSC* human bone marrow-derived mesenchymal (stromal) stem cell, DIC differential interference contrast

We subsequently determined whether the observed phenotype requires prerequisite direct cell–cell contact. We repeated the experiment using hMSCs and the MCF7 cell line with the transwell culture system, where hMSCs were cultured in the upper chamber and MCF7 tumor cells were cultured in the lower chamber or vice versa. As shown in Fig. 2a, b, there were no detectable changes in hMSC morphology or growth pattern. In addition, we subsequently established a co-culture system in a 35 mm dish whereby MCF7 cells were grown as a droplet on one side of the dish and FaDu cells in another droplet on the opposing side of the dish. When both cell types became adherent, hMSCs were added. As shown in Fig. 2c, d, e, f, the niche-like morphology of hMSCs was only observed in hMSCs at the contact site with MCF7 cells, but not with FaDu cells, corroborating that the observed changes in hMSC morphology are cancer cell-line specific and require direct cell–cell contact between hMSCs and tumor cells.

### Gene expression analysis of hMSCs sorted from MCF7 and BT-20 co-cultures revealed enrichment for pathways related to bone formation

**Fig. 3:**
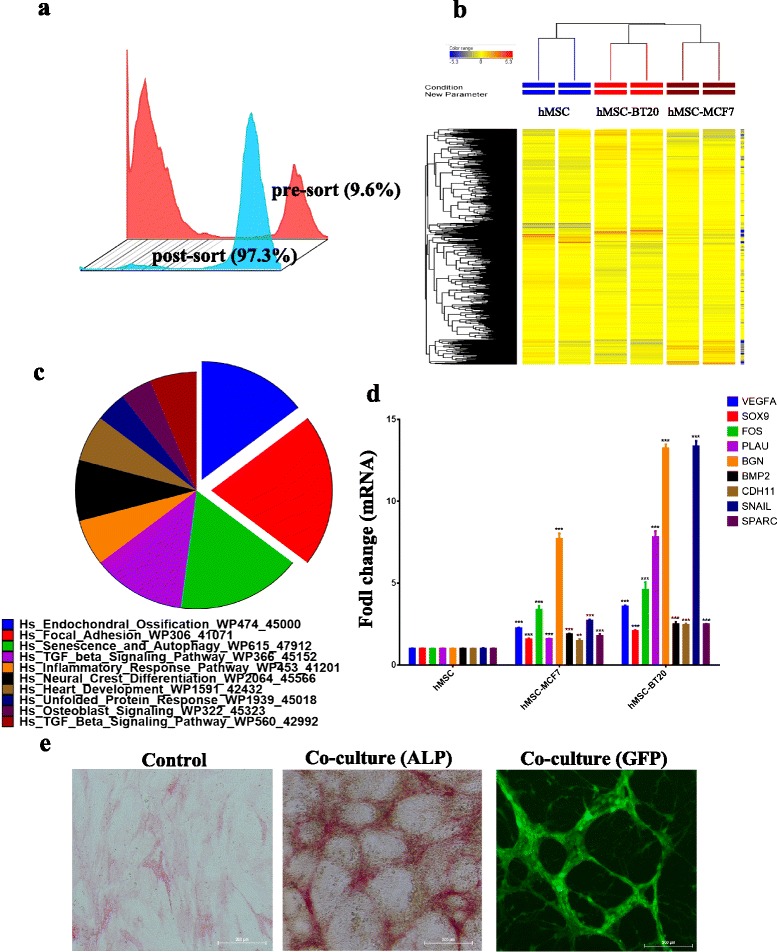
Gene expression analyses of hMSCs sorted following direct contact with either MCF7 or BT-20 cells in co-culture systems revealed enrichment for genes related to bone formation. hMSCs were co-cultured with MCF7 or with BT-20 tumor for 7 days. The cells were trypsinized and hMSCs were sorted using magnetic activated cell sorting technology: **a** efficiency of sorting. **b** Hierarchical clustering of control hMSCs (blue) or hMSCs sorted from MCF7 (*brown*) or BT-20 (*orange*) co-cultures. Each column represents one replica. Expression level of each gene in a single sample is depicted according to the color scale. **c** Pie chart illustrating the distribution of the top 10 GO categories for the upregulated genes in sorted hMSCs from both MCF7 and BT-20 co-cultures. The pie section size corresponds to fold enrichment. **d** qRT-PCR validation of selected genes from the microarray data. Data are presented as mean ± standard error, *n* = 6. **P <0.005; ***P <0.0005. **e** ALP staining for control hMSCs (*left*) or hMSCs following co-culture with MCF7 (day 7) (*middle*). Representative photomicrograph of hMSCs (GFP-labeled) in co-culture with MCF7 (*right*). *ALP* alkaline phosphatase, *GFP* green fluorescent protein, *hMSC* human bone marrow-derived mesenchymal (stromal) stem cell, GO gene ontology

To examine for the molecular changes induced in the niche-like structures formed by hMSCs co-cultured with MCF7 or BT-20 cells, hMSCs were negatively sorted using immune-magnetic beads (magnetic activated cell sorting) tagged with anti-human epithelial specific antigen (ESA; EpiCAM) of co-cultured cell populations. The procedure was repeated twice to increase the purity of sorted hMSCs. As shown in Fig. 3a, we were able to isolate hMSCs from the co-culture with very high purity (>97 %). Global gene expression analysis was determined using the Agilent® Human SurePrint microarray chip (G3 Human GE 8 × 60 k). We detected 218 upregulated genes and 92 downregulated genes in hMSCs sorted from MCF7 and BT-20 co-cultures compared with control hMSCs. As shown in Fig. 3b, hierarchical clustering revealed closer clustering of hMSCs sorted from MCF7 and BT-20 co-cultures compared with control hMSCs. Functional categories of the upregulated genes revealed significant enrichment for gene categories of bone formation: endochondral ossification, focal adhesion, osteoblast signaling, and transforming growth factor beta (TGF-β) signaling (Fig. 3c; Additional files 2 and 3). Gene expression of a selected number of genes from the microarray data (VEGFA, SOX9, FOS, PLAU, BGN, BMP2, CDH11, SNAI1, and SPARC) was subsequently validated using qRT-PCR (Fig. 3d). Concordant with the microarray data, ALP staining of hMSCs co-cultured with MCF7 revealed a remarkable increase in ALP activity (Fig. 3e middle panel), and ALP staining was restricted to the hMSCs and no ALP staining was detectable in cancer cells (compare Fig. 3e middle and right panels).

### Pharmacological inhibition of FAK, MAPKK, and actin polymerization completely abrogated niche formation by hMSCs

**Fig. 4:**
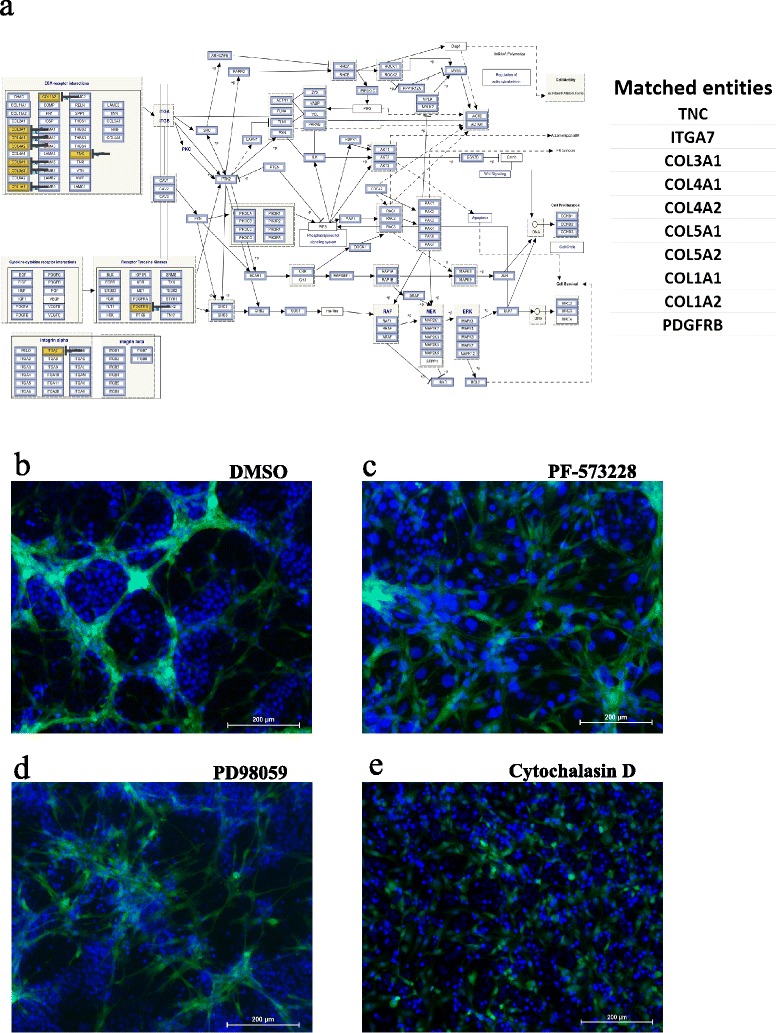
Pharmacological inhibition of FAK, MAPKK, and actin polymerization completely abrogated hMSC morphological changes. **a** The FAK pathway was among the top upregulated pathways in hMSCs following co-culture with MCF7 or BT-20 cancer cells. Matched entities are highlighted on the chart. Morphological changes in hMSCs following: disruption of the hMSC niche structure through pharmacological inhibition of **c** FAK (5 μM, PF-573228; Sigma), **d** MAPKK (5 μM, PD98059; Sigma) or **e** actin polymerization (1 μM, cytochalasin D; Sigma) compared with dimethyl sulfoxide (DMSO)-treated controls. **b**. DAPI was used to stain nuclei. All inhibitors were added on day 0 except for MAKK inhibitor which was added on day 0, and then co-cultures were supplemented with fresh inhibitor on day 2

Pathway analysis of upregulated genes in sorted hMSCs revealed multiple enriched intracellular signaling pathways. FAK (*P* = 1.3 × 10^−7^) and MAPKK (*P* = 0.014) were very prominent (Fig. 3c; Additional file 3). Upregulated genes in the FAK pathway are highlighted and are listed in Fig. 4a (right panel). To assess whether FAK and MAPKK signaling are involved in induction of morphological niche-like changes of hMSC in co-cultures, hMSCs and MCF7 cells were co-cultured as above in the presence of PF-573228 (FAK inhibitor, 5 μM), PD98059 (MAPKK inhibitor, 2 × 5 μM), or DMSO control. As shown in Fig. 4b, c, d, pharmacological inhibition of FAK or MAPKK abolished the niche-like morphological changes of hMSCs. We also postulated that the niche-like formation of hMSCs involves reorganization in the cellular cytoskeleton. To test this hypothesis, cytochalasin D (potent inhibitor of actin polymerization, 1 μM) was added to the co-cultures. As shown in Fig. 4e, inhibiting actin polymerization completely abolished the niche-like morphological changes in hMSCs.

### High expression of CDH1 and low expression of IL-1β by tumor cells are associated with hMSC niche-like formation

**Fig. 5:**
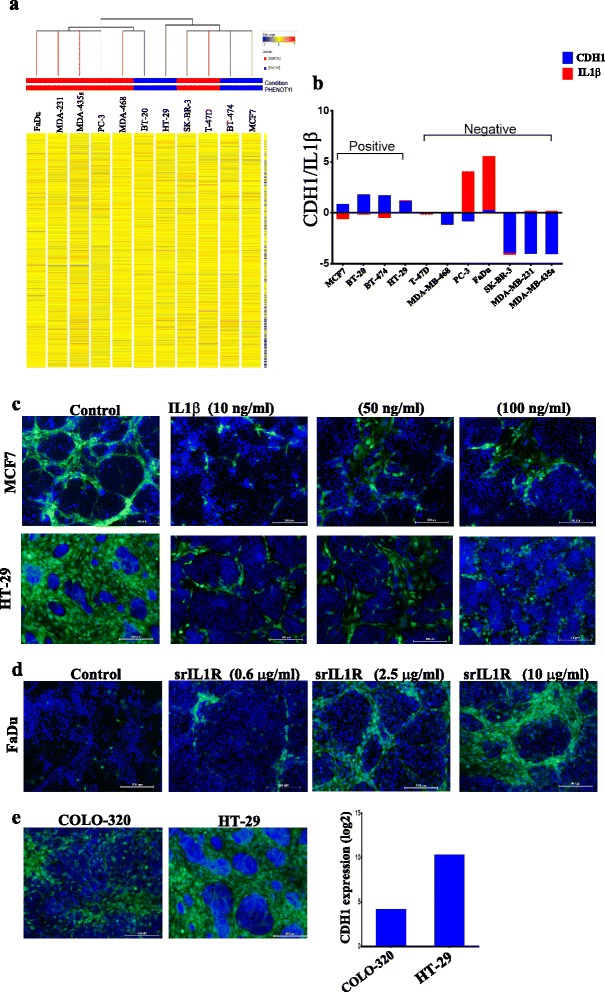
High expression of CDH1 and low expression of IL-1β by tumor cells are associated with induction of hMSC morphological changes. **a** Clustering analysis performed on basal gene expression of the employed cancer cell lines indicated close clustering for the cancer cell lines that induce morphological changes in hMSCs (positive cell lines, *blue*; MCF7, BT-474, HT-29, and BT-20) relative to cancer cell lines that do not induce morphological changes in hMSCs (negative cell lines, *red*). **b** gene expression data for CDH1 and IL-1β in cancer cell lines retrieved from microarray data. **c** Complete abrogation of hMSC morphological changes when co-cultured with MCF7 (*upper panels*) or HT-29 (*lower panels*) in the presence of increasing dose of IL-1β. **d** Dose-dependent reversal into hMSC niche formation when co-cultured with FaDu cells alone or in the presence of the indicated concentrations of soluble recombinant IL-1 receptor (srIL-1R). Images were captured on day 7 and DAPI was used to stain nuclei. **e** Lack of hMSC niche formation when co-cultured with COLO-320 cells (CDH1 negative/IL-1β negative) compared with the HT-29 cells (CDH1 positive/IL-1β negative). *CDH1* epithelial cadherin type 1, *IL* interleukin

To identify the molecular factors in tumor cells that promote or inhibit niche-like morphological changes in hMSCs, gene expression datasets for the cell lines utilized in the current study were retrieved from the gene expression omnibus [GEO:GSE36133] and imported into GeneSpring GX. Clustering analysis revealed close clustering of MCF7, BT-474, T-47D, and SKB-R3, followed by HT-29 and BT-20 (Fig. 5a). We subsequently grouped tumor cell lines into positive cell lines that induced hMSC morphological changes and niche-like formation (MCF7, BT-20, HT-29, and BT-474) and negative cell lines that did not induce morphological changes (FaDu, MDA-MB-231, MDA-MB-435s, PC-3, MDA-MB-468, SKB-R3, and T-47D). When comparing differentially expressed genes between the two cell groups, we observed CDH1 to be among the top genes highly expressed by the positive tumor group (Fig. 5b; Additional file 4). On the other hand, elevated expression of IL-1β was observed in several of the negative tumor group (Fig. 5b). To determine the inhibitory effects of IL-1β on morphological changes in hMSCs, hMSCs were co-cultured with MCF7 or HT-29 cells in the presence of vehicle or increasing dose of recombinant human IL-1β. As shown in Fig. 5c, significant inhibition of morphological changes and niche-like formation in hMSCs was observed. As a corollary, we investigated the effect of inhibiting IL-1β signaling in the co-culture system on hMSC niche-like formation. For this experiment, FaDu cells were chosen because they expressed the highest levels of IL-1β (Fig. 5b). As shown in Fig. 5d, a dose-dependent reversal into niche-like formation in hMSCs was observed when FaDu cells were co-cultured with hMSCs in the presence of increasing concentrations of soluble recombinant IL-1 receptor (srIL-1R). Interestingly, the niche-like formations observed in FaDu-hMSCs co-cultures in the presence of srIL-1R was indistinguishable from that observed in MCF7-hMSC co-cultures (Fig. 1). Therefore, our data suggest that tumor-derived IL-1β is a negative regulator of hMSC niche formation. In order to assess the role of CDH1 in niche formation, we co-cultured hMSCs with COLO-320 (which lacks CDH1 expression) or HT-29 (which expresses high levels of CDH1) and studied the development of the niche-like formation. Data presented in Fig. 5e showed lack of niche formation in COLO-320–hMSC co-cultures compared with the HT-29–hMSC co-cultures, suggesting that CDH1 is potentially involved in promoting niche formation possibly through promoting homotypic cell adhesion.

### Transfer of cellular components from cancer cells to hMSCs with no evidence of cell fusion during co-culture

**Fig. 6:**
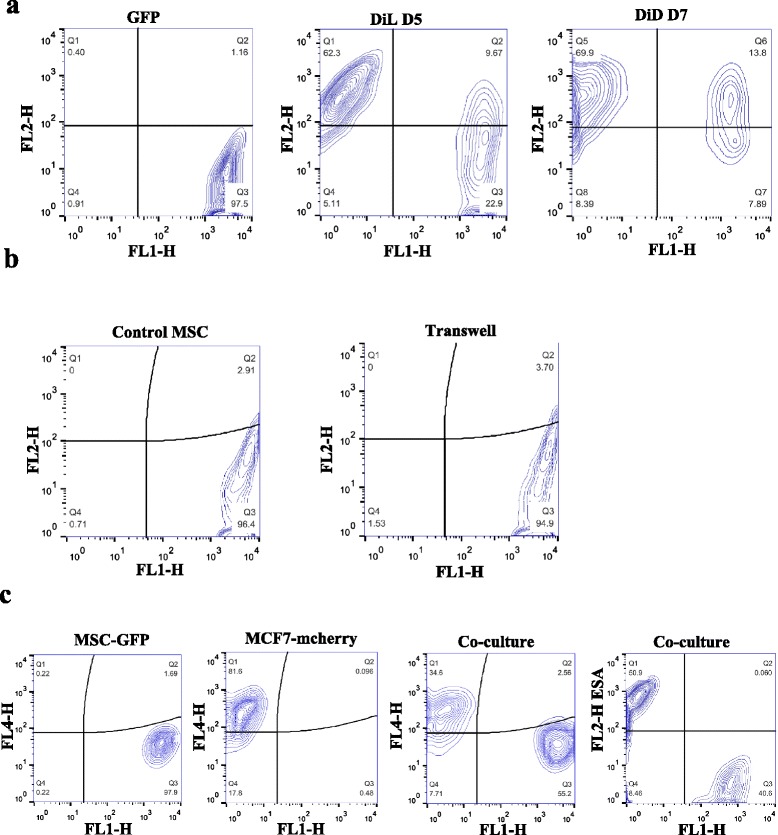
Transfer of cellular components from cancer cells to hMSCs but no evidence of cell fusion during co-culture. **a** hMSCs (GFP-labeled) were co-cultured with MCF7 labeled with DiL (for 5 days) or DiD (for 7 days) and subsequently were subjected to FACS analysis. **b** hMSCs were cultured alone (left) or were cultured in the upper chamber, while MCF7 cells labeled with DiD were cultured in the lower chamber and then cells were subjected to FACS analysis. **c** hMSCs (GFP-labeled, *panel 1*) or MCF7 (mcherry-labeled, *panel 2*) were cultured alone or were co-cultured (*panel 3*) and subjected to FACS analysis. Similarly, co-cultured hMSCs and MCF7 cells were stained for ESA and were subjected to FACS analysis. *ESA* epithelial specific antigen, *GFP* green fluorescent protein, *MSC* mesenchymal (stromal) stem cell

Data presented in Figs 1 and 2 suggest that the formation of hMSC niche-like morphological changes requires cell–cell contact. Cancer cells were therefore labeled with DiL or DiD lipophilic tracer dyes (which specifically label plasma membranes). Subsequently, hMSCs and cancer cells were co-cultured in direct contact (Fig. 6a) or separated using a transwell culture system (Fig. 6b). We observed noticeable transfer of cellular components from cancer cells to hMSCs in a time-dependent manner (~70 % on day 7), which occurred when the two cells types were co-cultured in direct contact (compare Fig. 6a middle and right with Fig. 6b right). In addition, fluorescence microscopy revealed transfer of microvesicles between fluorescence-labeled AT-MSCs and labeled MCF7 cells when co-cultured in direct contact (Additional file 5). A previous study reported that hMSCs were able to fuse with other cell types, such as epithelial cells, when co-cultured in vitro [21]. In order to address whether cellular fusion took place between hMSCs and cancer cells under our culture conditions, GFP-labeled hMSCs were co-cultured with mcherry-labeled MCF7 cells for 7 days. As shown in Fig. 6c, FACS analysis revealed no evidence of cellular fusion. Similarly, hMSCs from the co-cultures did not stain for ESA—again proving lack of cellular fusion between hMSCs and cancer cells during co-cultures (Fig. 6c right panel). Our data suggest that microvesicular transfer of cellular components from tumor cells to hMSCs is a possible mechanism of cellular communication mediating morphological changes of niche-like formation of hMSCs.

### Bidirectional influence on cell growth between hMSCs and cancer cells in co-cultures

**Fig. 7:**
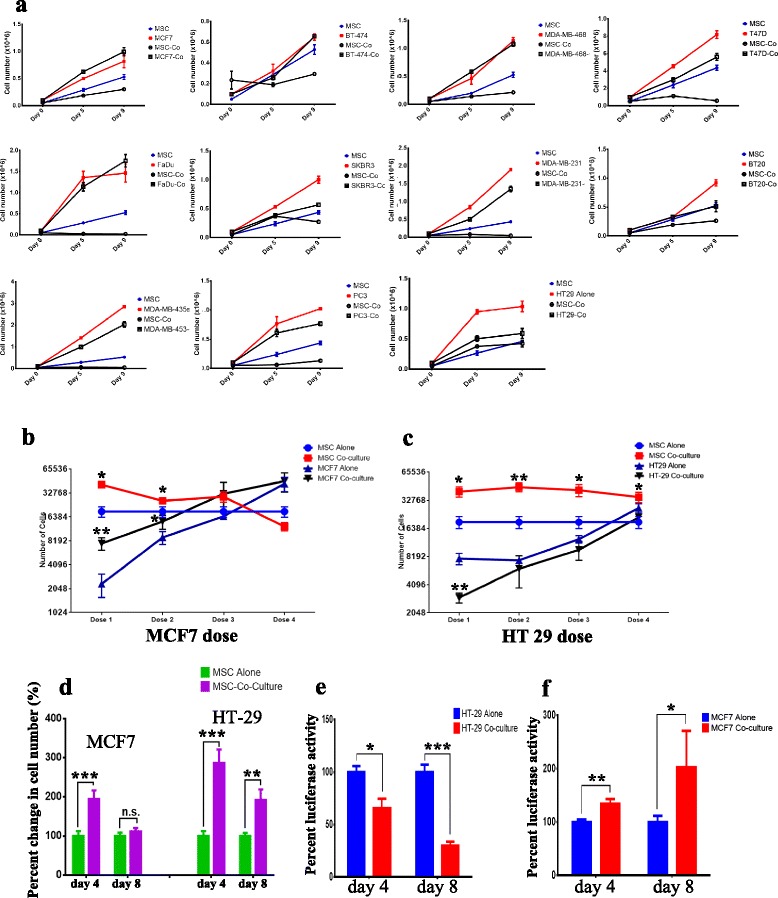
Bidirectional influence on cell growth between hMSCs and cancer cell lines in co-culture. **a** hMSCs were co-cultured with the indicated tumor cell lines, and on days 5 and 9 the number of hMSCs and cancer cells from either the co-cultures or from cells cultured alone were enumerated. Data presented as mean ± standard error (SE), *n* = 3. A fixed number of hMSCs (0.5 × 10^4^) was co-cultured with an increasing number of MCF7 cells (**b** 1.0 × 10^3^, 0.5 × 10^4^, 1.0 × 10^4^, 0.5 × 10^5^) or HT29 cells (**c** 1.0 × 10^3^, 0.5 × 10^4^, 1.0 × 10^4^, 0.5 × 10^5^) and the total number of hMSCs or tumor cells was enumerated on day 6. Data presented as mean ± SE, *n* = 6. **d** hMSCs (0.5 × 10^4^) were co-cultured with MCF7 (0.5 × 10^3^) or HT-29 (0.5 × 10^3^) cells and the relative hMSC number was enumerated using a SpectraMax/M5 fluorescence spectrophotometer plate reader. Data presented as mean ± SE. Relative number of **e** HT-29-Luc or **f** MCF7-Luc number from co-cultures was enumerated using luciferase assay on days 4 and 8. Data presented as mean ± SE, *n* = 6. *MSC* mesenchymal (stromal) stem cell; *P <0.05; **P <0.005; ***P <0.0005

We also examined the possible effects of hMSC niche formation on cancer cell growth. hMSCs were co-cultured with the indicated cancer cell lines, and on days 5 and 9 we employed FACS analysis to determine the percentage of hMSCs and cancer cells in the co-culture and automated cell counting (Vi-CELL) to determine the total number of cells. The data presented in Fig. 7 demonstrate that in most cases (except MCF7) the cancer cell number was usually less when co-cultured with hMSCs compared with cancer cells cultured alone (Fig. 7a). We also observed a significant reduction in the number of hMSCs co-cultured with cancer cells that did not induce niche formation (i.e. FaDu). Under certain conditions, we observed a slight increase in hMSC number when co-cultured with cancer cells (i.e. HT-29).

**Fig. 8:**
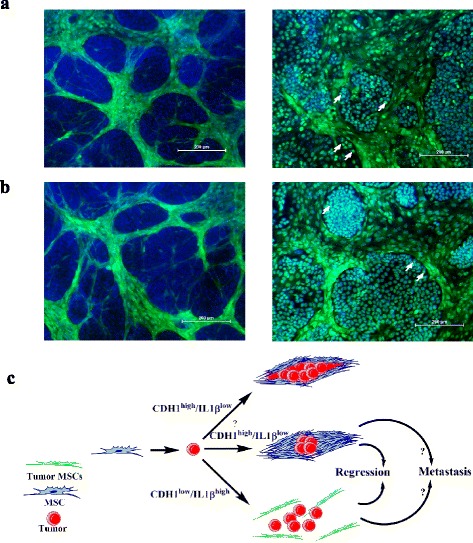
Increased proliferation of MCF7 when co-cultured with hMSCs. MCF7 cells were co-cultured with hMSCs, and on day 7 cells were fixed and stained with the proliferation markers **a** Ki67 or **b** PCNA. Control staining is shown on each left panel. *Arrowhead* indicates cells with high Ki67 or PCAN staining. **c** Schema depicting possible interaction between hMSCs and tumor cells. Tumor cells with high CDH1 and low IL-1β will promote niche formation which might inhibit or promote tumor growth. Tumors with low CDH1 and high IL-1β will lead to rapid decline in hMSC numbers, and vice versa the number of tumor cells will decline as well and the hMSC niche will not form. Selective pressure exerted by MSCs could lead to regression of primary tumors and the acquisition of metastatic phenotype by tumor cells. *GFP* green fluorescent protein, *IL* interleukin, *MSC* mesenchymal (stromal) stem cell

We also determined whether the ratio of cancer cells to hMSCs is a factor in determining the outcome of co-cultures. We cultured a fixed number of hMSCs (0.5 × 10^4^) with an increasing number of MCF7 (1.0 × 10^3^, 0.5 × 10^4^, 1.0 × 10^4^, 0.5 × 10^5^) or HT-29 (1.0 × 10^3^, 0.5 × 10^4^, 1.0 × 10^4^, 0.5 × 10^5^) tumor cells. The number of hMSCs and cancer cells on day 6 was determined. When a low number of MCF7 cells was employed, we observed a significant increase in the number of hMSCs compared with hMSCs cultured alone (Fig. 7b). Similarly, time-lapse live cell imaging microscopy revealed dynamic interaction between hMSCs and MCF7, with higher mitotic activity in hMSCs co-cultured with MCF7 cells (Additional file 6). On the other hand, a higher number of MCF7 cells was observed in the hMSC–MCF7 co-cultures compared with MCF7 cultured alone (Fig. 7b). When examining the co-cultures of hMSCs with HT-29 cells, we also observed an increase in the number of hMSCs (Fig. 7c). In contrast, the number of HT-29 tumor cells decreased when co-cultured with hMSCs, suggesting a possible anti-tumor effect of hMSCs. In order to further confirm these findings, we utilized GFP-labeled hMSCs and MCF7 or HT-29 cells engineered to express the firefly luciferase gene. As shown in Fig. 7d, e, f, a rapid increase in hMSC number was observed when co-cultured with MCF7 or HT-29 cells which was associated with a significant decline in the number of HT-29 cells and an increase in the number of MCF7 cells. Concordant with data presented in Fig. 7a, b, f, Ki67 and PCNA staining (markers for cell proliferation) of MCF7–hMSC co-cultures revealed high expression of ki67 and PCNA in MCF7 cells that are in close contact with the hMSC niche formation (Fig. 8a, b).

## Discussion

Recent years have witnessed increased interest in studying the cancer tumor microenvironment and its contribution to tumor progression, invasion, and metastasis. However, the precise role of MSCs—an important component of the cancer tumor microenvironment—remains not fully understood. In the current study, we have utilized several cellular and molecular approaches to investigate the dynamic interaction between hMSCs and cancer cells. We employed several cancer cell lines representing breast, prostate, colon, head and neck, and melanoma. Our data revealed that hMSC interaction with cancer cells results in cytoskeletal and morphological reorganization leading to the formation of niche-like structures that exert variable effects on cancer cell growth. We also observed that cancer cell expression of CDH1/IL-1β is predictive for the presence of significant biological effects of hMSCs on tumor cells.

We observed that hMSCs exerted anti-tumor effects, evident by a significant decline in the number of tumor cells when co-cultured with hMSCs (Fig. 7a). Cell tracking and live imaging microscopy revealed a rapid increase in the numbers of hMSCs when they encountered cancer cells (Fig. 7a, c, d; Additional file 6), suggesting that hMSCs may act as the first line of defense against the transformed cells. Concordant with our hypothesis, one previous study has demonstrated a protective role for skin-derived fibroblasts in regulating transformed keratinocytes in vivo [22].

When hMSCs were co-cultured with cancer cells with aggressive phenotype (such as FaDu, PC-3, or MDA-MB-231), we observed a rapid decline in the number of hMSCs suggesting that the behavior of hMSCs is largely dependent on the nature of cancer cells, in particular their inflammatory status. Our data revealed that the decline in hMSC number occurred when co-cultured with cancer cells expressing high levels of IL-1β.

We previously reported that when hMSCs are exposed to IL-1β they acquire a proinflammatory phenotype and exhibit a decline in their multilineage differentiation potential [20]. In the current study, when hMSCs were co-cultured with IL-1β producing cancer cells, hMSCs became proinflammatory cells and their number declined. The role of IL-1β was demonstrated by the complete reversal in hMSC niche formation when IL-1β signaling was blocked during hMSC–FaDu co-cultures (Fig. 5d).

We observed that hMSCs formed niche-like structures when co-cultured with cancer cells expressing high levels of CDH1 and lacked IL-1β (Fig. 7h). Interestingly, our data are in line with a published report suggesting that the inhibitory ability of hMSCs on tumor cell growth is mediated through binding to E-cadherin present on tumor cells [8]. Concordant with this, niche formation was not observed when hMSCs were co-cultured with the COLO-320 cells (which lack CDH1 expression) compared with the HT-29 cells (which express high levels of CDH1), suggesting a possible role for CDH1 in promoting niche formation, possibly through facilitating homotypic cell adhesion.

While we have not characterized the nature of the hMSC niche-like formation under all co-culture conditions, the hMSC niches formed in the presence of MCF7 and BT-20 cells revealed differentiation into bone-forming osteoblastic cells, evident by microarray analysis and positive ALP expression (Fig. 3c, e). hMSC niche-like formations were dependent on FAK and MAPKK signaling because pharmacological inhibition of both pathways completely abrogated the niche-like formations. Interestingly, Navab et al. [23] have recently identified a prognostic gene expression signature derived from CAFs isolated from lung cancer patients and reported that FAK and MAPK are two major intracellular signaling pathways activated in CAFs, which collectively suggest the clinical relevance of our findings.

One interesting finding in our study is the transfer of cellular components from cancer cells to hMSCs. The transfer of membrane-derived vesicles has been reported previously in various biological systems as a mode of communication between adjacent cells [24, 25]. It is plausible that changes in hMSC phenotype when co-cultured with cancer cells are mediated via tumor-derived factors that induce hMSC differentiation. Interestingly, while morphologically similar, the niche-like formations in hMSCs co-cultured with HT-29 did not stain positive for ALP (Additional file 7) and exerted negative effects on cancer cell growth (Fig. 7a, e). Similarly, both HT-29 and MCF7 co-cultured with hMSCs had less ability to close the gap during the wound healing assay (Additional file 8). Our current hypothesis is that the niche-like formation of hMSCs plays a role in controlling cancer cell growth and spread. Our data are in agreement with recently published data implicating pericytes, which are related ontologically to MSCs, in preventing metastasis in vivo [7].

We observed that cell growth of MCF7 increased when co-cultured with hMSCs. Similar to our data, Karnoub et al. [4] showed that MCF7 is the only breast cancer cell line to have significant increase in tumor growth when implanted with MSCs in vivo. The biological differences between niche-like formation of hMSCs in response to MCF7 versus that induced by HT-29 cells are not known. However, this may be related to differences in multiple activated genetic pathways in MCF7 versus HT-29 as revealed by differences in their molecular signature (Additional file 9). We propose a working hypothesis in which MSCs interact with cancer cells and the outcome of this dynamic interaction is dependent on the expression of CDH1/IL-1β by cancer cells (Fig. 8c). MSCs exert inhibitory effects on cancer cell growth. However, it is possible that this interaction may also exert selection pressure on certain types of cancer cells leading to increased cancer cell growth and possibly metastases (Fig. 8c). Detailed molecular analysis of this hypothesis remains to be determined.

## Conclusions

Our data revealed dynamic bidirectional interaction between hMSCs and tumor cells. Data also show that this interaction is dependent on the nature rather than the type of tumor cells and that CDH1 and IL-1β expression by tumor cells are key factors in determining the outcome of hMSC–tumor cross-talk.

## Additional files

Additional file 1:
**Is Table S1 presenting primer sequences used for qRT-PCR.** (DOCX 16 kb) Additional file 2:
**Is Table S2 presenting upregulated genes in sorted MSCs from MCF7 and BT-20 co-cultures.** (XLSX 19 kb) Additional file 3:
**Is Table S3 presenting the top enriched pathways in upregulated genes in sorted MSCs from MCF7 and BT-20 co-cultures.** (XLSX 12 kb) Additional file 4:
**Is Table S4 presenting genes differentially expressed between positive and negative tumors.** (XLSX 31 kb) Additional file 5:
**Is Figure S1 showing transfer of cellular components between hMSCs and cancer cells. **(DOCX 214 kb) Additional file 6:
**Is Movie S1 showing time-lapse microscopy of MCF7 cells (** ***red*** **) co-cultured with hMSCs (** ***green*** **).** Images were taken every 30 minutes using Nikon® BioStation IM-Q. (AVI 752 kb) Additional file 7:
**Is Figure S2 showing ALP staining for hMSC–HT-29 co-culture.** (DOCX 409 kb) Additional file 8:
**Is Figure S3 showing the scratch assay for HT-29 and MCF7, alone or co-cultured with hMSCs.** (DOCX 308 kb) Additional file 9:
**Is Table S5 presenting the top pathways for MCF7 versus HT-29.** (XLSX 15 kb)

## Abbreviations

AKT: RAC-alpha serine/threonine-protein kinase
ALP: Alkaline phosphatase
AT-MSC: Adipose tissue-derived mesenchymal (stromal) stem cell
BGN: Biglycan
BMP2: Bone morphogenetic protein 2
BSA: bovine serum albumin
CAF: Carcinoma-associated fibroblast
CDH1: Epithelial cadherin type 1
CDH11: Cadherin-11
DAPI: 4′,6-Diamidino-2-phenylindole
DMEM: Dulbecco’s modified Eagle’s medium
DMSO: Dimethyl sulfoxide
EDTA: Ethylenediamine tetraacetic acid
EGF: Epidermal growth factor
ESA: Epithelial specific antigen
FACS: Fluorescence-activated cell sorting
FAK: Focal adhesion kinase
FBS: Fetal bovine serum
FOS: c-Fos proto-oncogene
GFP: Green fluorescent protein
hMSC: Human bone marrow-derived mesenchymal (stromal) stem cell
IL: Interleukin
Ki67: Marker of proliferation Ki-67
MAPKK: Mitogen-activated protein kinase kinase
MSC: Mesenchymal (stromal) stem cell
PBS: Phosphate-buffered saline
PLAU: Urokinase-type plasminogen activator
qRT-PCR: Quantitative real-time polymerase chain reaction
SNAI1: Zinc finger protein
SOX9: SRY (sex determining region Y) Box 9
SPARC: Secreted protein, acidic, cysteine-rich (osteonectin)
srIL-1R: Soluble recombinant IL-1 receptor
TERT: Telomerase reverse transcriptase
TGF-β: Transforming growth factor beta
VEGFA: Vascular endothelial growth factor A
