# Supplementary material for: CDH1 and IL1-beta expression dictates FAK and MAPKK-dependent cross-talk between cancer cells and human mesenchymal stem cells
Source: Stem Cell Res Ther. 2015 Jul 24;6(1):135. doi: 10.1186/s13287-015-0123-0 (PMC4533790; doi:10.1186/s13287-015-0123-0)
Supplement: Additional file 1: — Is Table S1 presenting primer sequences used for qRT-PCR. (DOCX 16 kb) [file 13287_2015_123_MOESM1_ESM.docx]

| Table S1: Primer sequences used for qRT-PCR | | |
| --- | --- | --- |
| **No.** | **Name** | **Sequence** |
| **1** | **CDH1**  **F**  **R** | 5` GGCGGAGAAGAGGACCAGGACT 3`  5` CATCGGGATTGGCAGGGCGG 3` |
| **2** | **VEGFA**  **F**  **R** | 5` ACAACAAATGTGAATGCAGACCA 3`  5` TACCGGGATTTCTTGCGCTT 3` |
| **3** | **SOX9**  **F**  **R** | 5` cccttcaacctcccacacta 3`  5` tggtggtcggtgtagtcgta 3` |
| **4** | **FOSB**  **F**  **R** | 5` GCGCCGGGAACGAAATAAAC 3`  5` CAACTGATCTGTCTCCGCCT 3` |
| **5** | **PLAU**  **F**  **R** | 5` ACTCCAAAGGCAGCAATGAAC 3`  5` GTGCTGCCCTCCGAATTTCT 3` |
| **6** | **BGN**  **F**  **R** | 5` TCCACGACAACCGCATCCGC 3`  5` AGGCCATCGAAGGCTCCAGGT 3` |
| **7** | **BMP2**  **F**  **R** | 5` CTGCGGTCTCCTAAAGGTCG 3`  5` AGCAGCAACGCTAGAAGACA 3` |
| **8** | **CDH11**  **F**  **R** | 5` ACCAAGCCACTTTCCAACCA 3`  5` ACGCCTGCTGTGTTATCTCG 3` |
| **9** | SNAI1  **F**  **R** | 5` CCAGTGCCTCGACCACTATG 3`  5` CTGCTGGAAGGTAAACTCTGGA 3` |
| **10** | **SPARC**  **F**  **R** | 5` gaggaaaccgaagaggagg 3`  5` ggggtgttgttctcatccag 3` |
