# Supplementary material for: CDH1 and IL1-beta expression dictates FAK and MAPKK-dependent cross-talk between cancer cells and human mesenchymal stem cells
Source: Stem Cell Res Ther. 2015 Jul 24;6(1):135. doi: 10.1186/s13287-015-0123-0 (PMC4533790; doi:10.1186/s13287-015-0123-0)
Supplement: Additional file 5: — Is Figure S1 showing transfer of cellular components between hMSCs and cancer cells. (DOCX 214 kb) [file 13287_2015_123_MOESM5_ESM.docx]

**
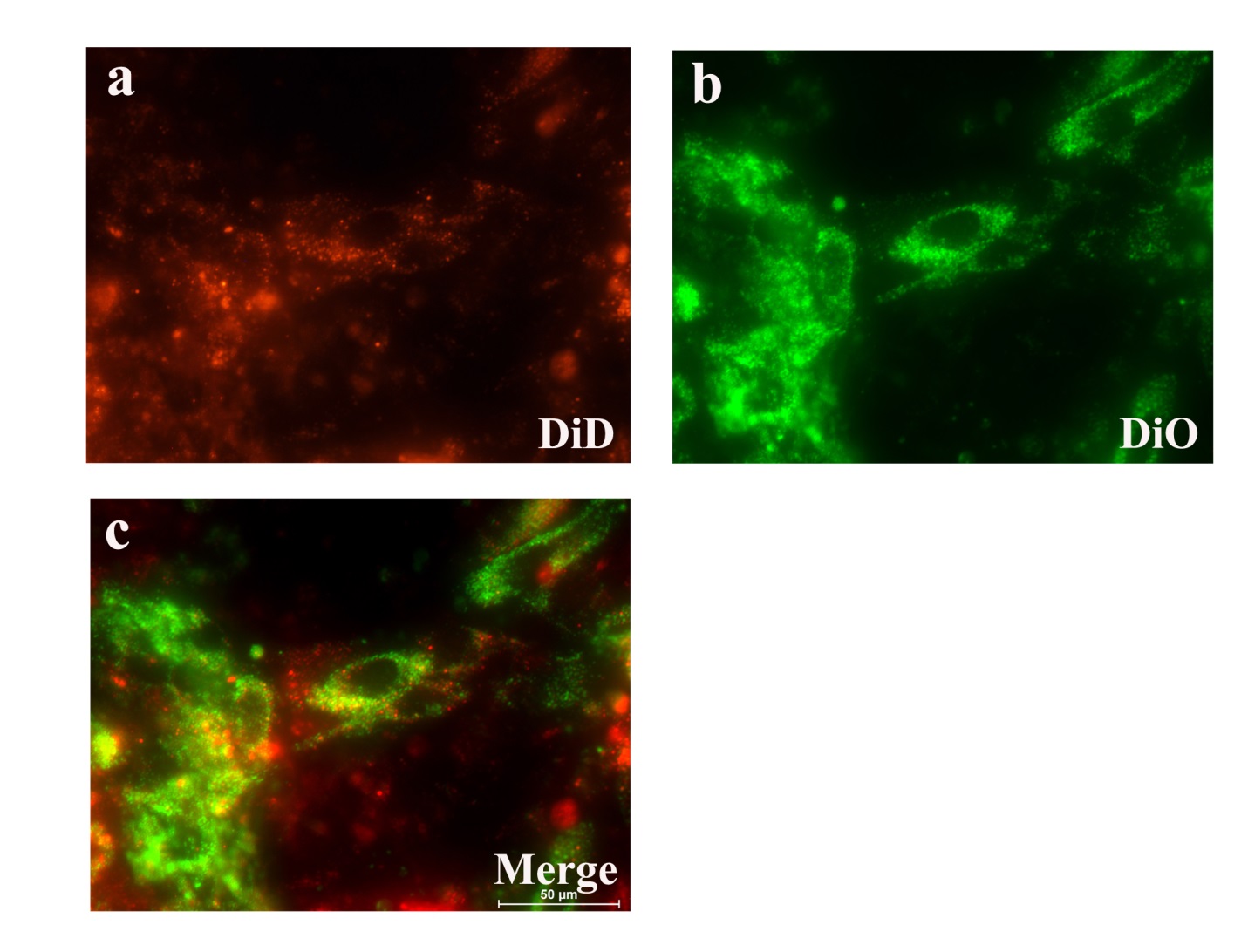
**

**Figure S1, Related to figure 6. Transfer of cellular components between hMSCs and cancer cells.** MCF7 cells were labeled with DiD (a, red) while ATMSCs were labeled with DiO (b, green) and were co-culturd for 7 days. (c) Merge of green and red channels showing niche formation by ATMSCs and the uptake of microvesicles derived from MCF7 cells (arrowheads) by ATMSCs. Imaging was conducted using 20x objective using Nikon® ECLIPSE Ti-U inverted fluorescence microscope.
