# Supplementary material for: CDH1 and IL1-beta expression dictates FAK and MAPKK-dependent cross-talk between cancer cells and human mesenchymal stem cells
Source: Stem Cell Res Ther. 2015 Jul 24;6(1):135. doi: 10.1186/s13287-015-0123-0 (PMC4533790; doi:10.1186/s13287-015-0123-0)
Supplement: Additional file 7: — Is Figure S2 showing ALP staining for hMSC–HT-29 co-culture. (DOCX 409 kb) [file 13287_2015_123_MOESM7_ESM.docx]

**
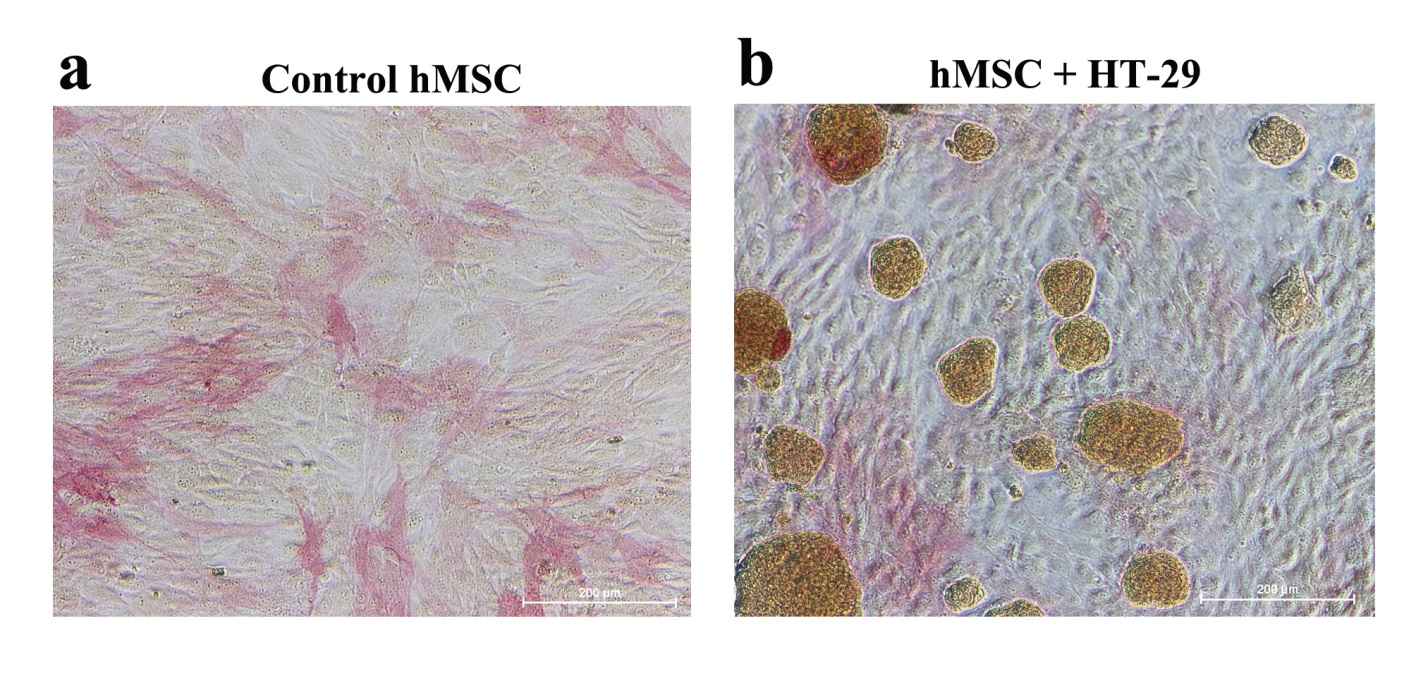
**

**Figure S2, Related to Figure 3. ALP staining for hMSC-HT-29 coculture.** hMSC were cultured alone (a) or with HT-29 (b) then on day7, ALP staining was performed and imaging was conducted using 4x magnification using Nikon® ECLIPSE Ti-U inverted fluorescence microscope.
