# Supplementary material for: CDH1 and IL1-beta expression dictates FAK and MAPKK-dependent cross-talk between cancer cells and human mesenchymal stem cells
Source: Stem Cell Res Ther. 2015 Jul 24;6(1):135. doi: 10.1186/s13287-015-0123-0 (PMC4533790; doi:10.1186/s13287-015-0123-0)
Supplement: Additional file 8: — Is Figure S3 showing the scratch assay for HT-29 and MCF7, alone or co-cultured with hMSCs. (DOCX 308 kb) [file 13287_2015_123_MOESM8_ESM.docx]

**
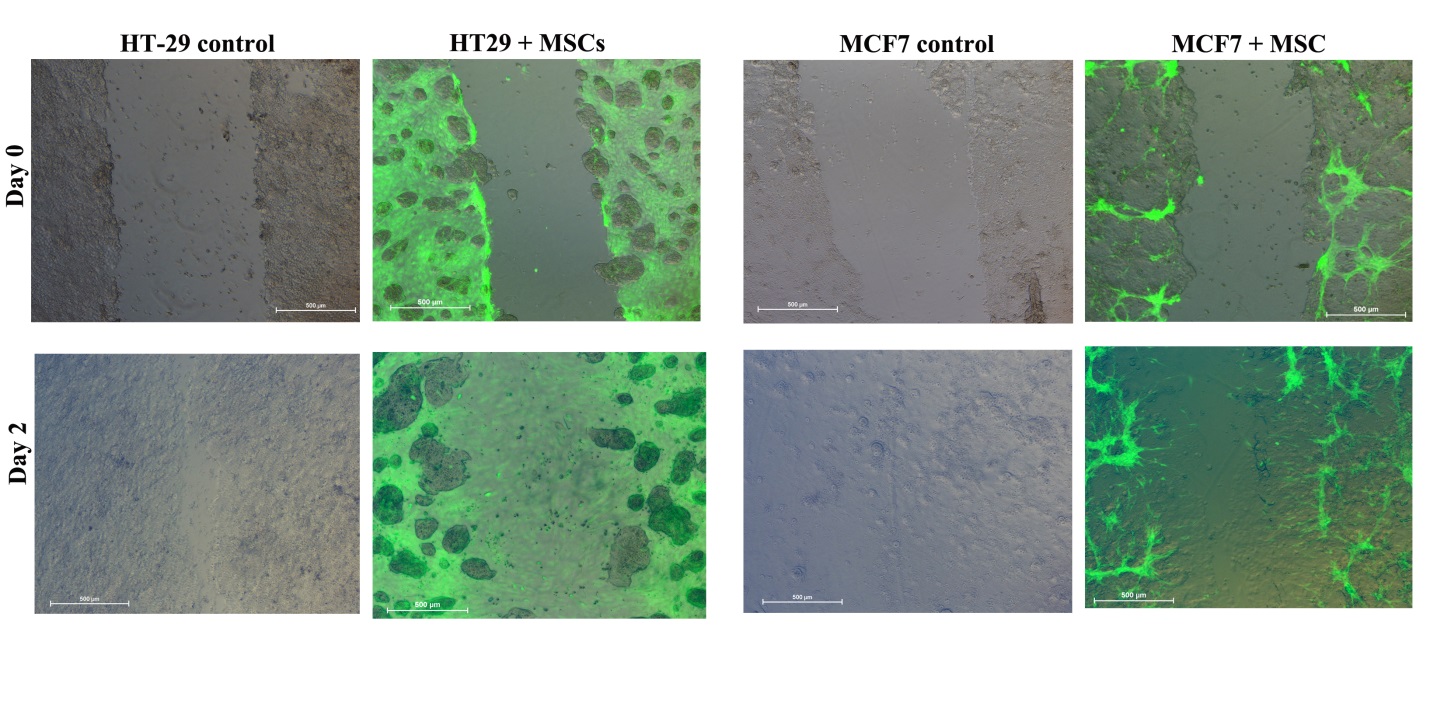
**

**Figure S3, Related to Figure 7.** **Scratch assay for HT-29, MCF7, alone or cocultured with hMSCs.** HT-29 and MCF7 cells were cultured alone or were co-cultured with hMSC and on day7, scratch assay was performed using p200 pipet tip. Scratch area was imaged on days 0 and day2 using 4x magnification using Nikon® ECLIPSE Ti-U inverted fluorescence microscope. Data are representative of at least 3 replicas.
